# Supplementary figures and images for: The high price of equity in pulse oximetry: A cost evaluation and need for interim solutions
Source: PLOS Digit Health. 2024 Sep 30;3(9):e0000372. doi: 10.1371/journal.pdig.0000372 (PMC11441667; doi:10.1371/journal.pdig.0000372)

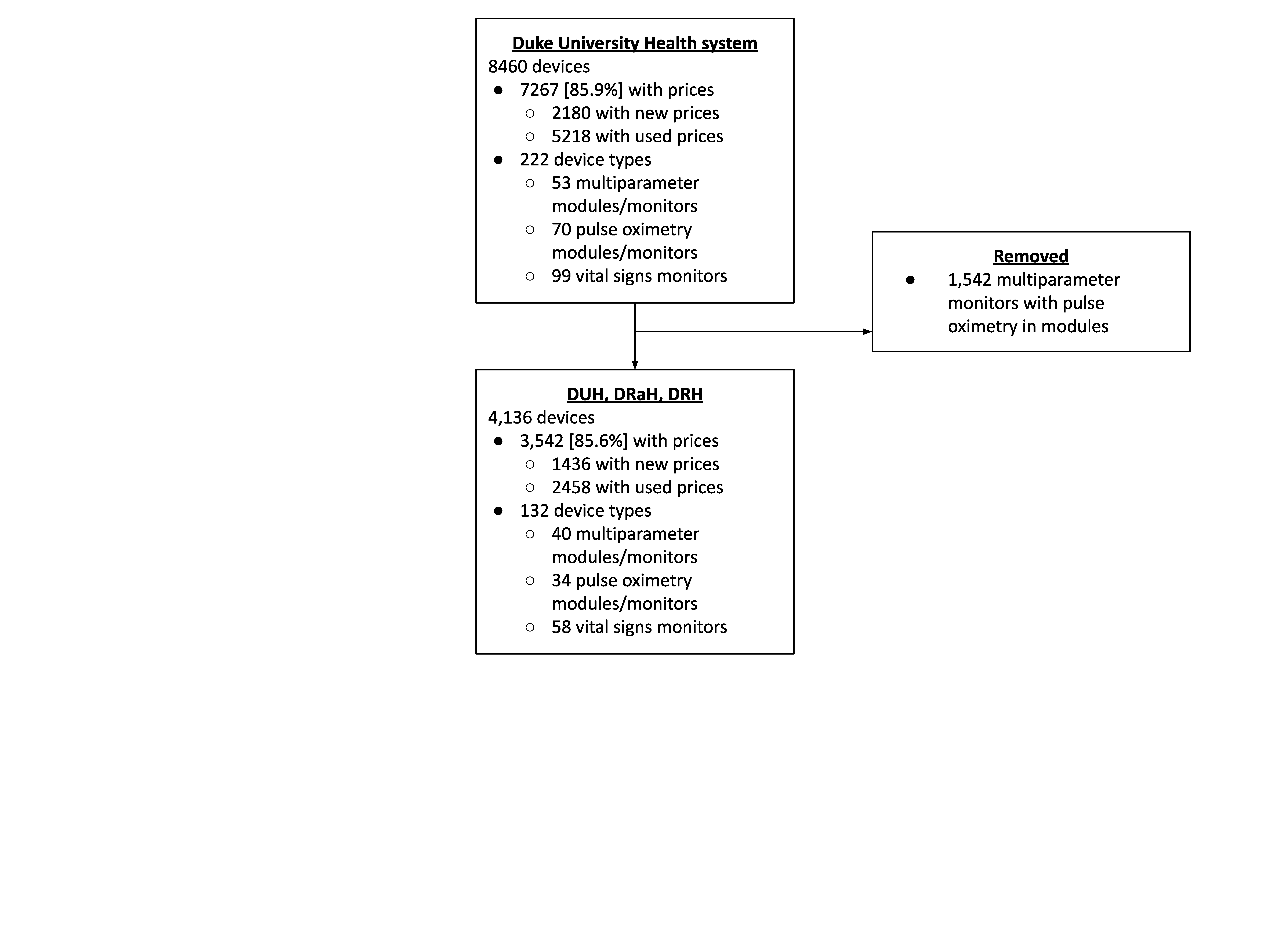

Supplement: S1 Fig — Flow diagram for all devices at Duke University Health System, focused on in-hospital devices for acute care. Note that 1,542 multiparameter monitors were excluded as they would not need to be replaced, as their pulse oximetry was measured in modules. Furthermore, note that for DUH, DRaH, and DRH, the sum of new (1436) and used equipment with prices (2458) is higher by 336 (3,542 with prices), as 336 devices had both new and used prices. (TIFF) [file pdig.0000372.s001.tiff]

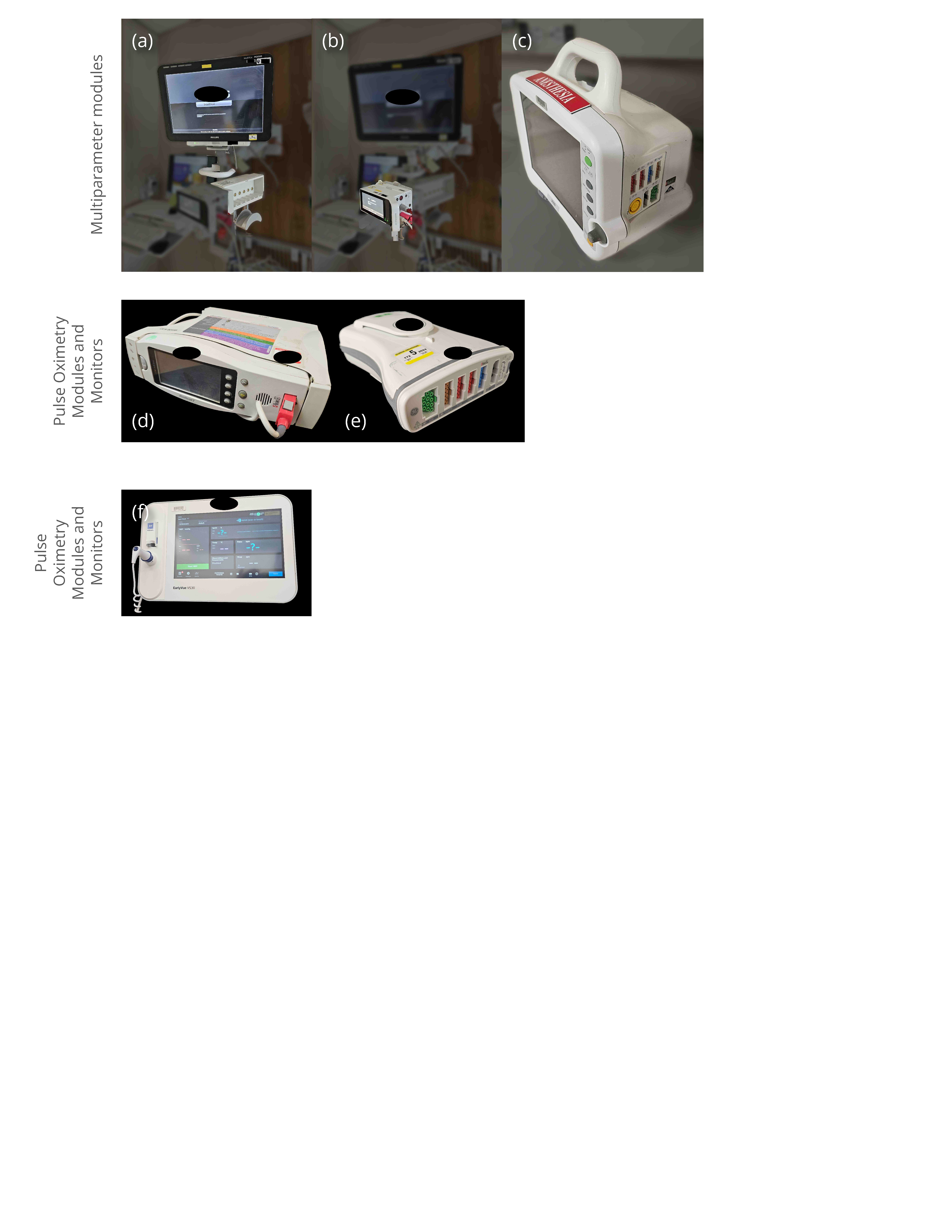

Supplement: S2 Fig — Example of devices referenced in Table 1. a. Multiparameter module: Philips MX800. b. Multiparameter module: Philips X3. c. Multiparameter module: GE Dash 3000. d. Pulse oximetry modules and monitors: Masimo Radical-7. e. Pulse oximetry modules and monitors: GE Patient Data Module. f. Vital signs monitors: Philips EarlyVue VS30. (TIFF) [file pdig.0000372.s002.tiff]
