# Supplementary material for: The high price of equity in pulse oximetry: A cost evaluation and need for interim solutions
Source: PLOS Digit Health. 2024 Sep 30;3(9):e0000372. doi: 10.1371/journal.pdig.0000372 (PMC11441667; doi:10.1371/journal.pdig.0000372)
Supplement: S1 Table — (DOCX) [file pdig.0000372.s003.docx]

## S1 Table. List of price estimates for devices

This is a list of prices for new and used devices found online. The top devices with percentage rates over 1% are included. A table with links is included online. An estimated replacement price is taken as the maximum of the new device price and the used device price divided by 75%. A 75% used price to new price ratio suggests that a hypothetical device that can be purchased used for $750 is estimated to have an MSRP of $1,000.

|  |  |  |  | **new device** | | **used device** | | **estimated replacement price** |
| --- | --- | --- | --- | --- | --- | --- | --- | --- |
| **Manufacturer** | **Category** | **Device model** | **% total** | **price** | **source** | **price** | **source** |  |
| Philips Healthcare | Multi-Parameter Module/Monitor | X2 M3002A | 11.48% |  |  | $ 1,250.00 | <https://www.dotmed.com/listing/bedside-monitor/philips/intellivue-x2-vital-sign-patient-monitor-m3002a-with-new-battery/3585485> | $ 1,666.67 |
| Philips Healthcare | Multi-Parameter Module/Monitor | MX450 | 11.34% |  |  | $ 5,937.00 | <https://www.medicalpriceonline.com/medical-equipment/philips/philips-intellivue-mx450-bedside-monitor/> | $ 7,916.00 |
| Philips Healthcare | Vital Signs Monitor | SureSigns VS4 | 6.42% | $ 6,857.00 | <https://www.altramedical.com/philips-suresigns-vs4-vital-signs-monitor/> | $ 1,336.00 | <https://bimedis.com/philips-suresigns-vs4-m360190> | $ 6,857.00 |
| Philips Healthcare | Multi-Parameter Module/Monitor | X3 | 5.51% |  |  | $ 4,375.00 | <https://bimedis.com/philips-intellivue-x3-m486733> | $ 5,833.33 |
| Masimo Corp | Pulse Ox Module/Monitor | Rad-7 | 5.28% | $ 2,149.00 | <https://mfimedical.com/products/masimo-radical-7-pulse-oximeter?variant=44752125582&currency=USD> |  |  | $ 2,149.00 |
| Philips Healthcare | Multi-Parameter Module/Monitor | MX800 | 5.24% |  |  | $ 9,994.00 | <https://www.dotmed.com/listing/bedside-monitor/philips/intellivue-mx800-icu-ccu-monitor/2684138> | $ 13,325.33 |
| Philips Healthcare | Multi-Parameter Module/Monitor | M3001A | 4.65% |  |  | $ 683.00 | <https://bimedis.com/philips-m3001a-m477338> | $ 910.67 |
| Philips Healthcare | Vital Signs Monitor | VS30 EarlyVue | 4.37% | $ 1,321.00 | <https://bimedis.com/philips-earlyvue-vs30-m600696> |  |  | $ 1,321.00 |
| Masimo Corp | Pulse Ox Module/Monitor | Rad-5v | 3.91% | $ 759.00 | <https://www.concordhealthsupply.com/Masimo-Rad-5v-Handheld-Pulse-Oximeter-p/mas-9197.htm> |  |  | $ 759.00 |
| Welch Allyn Inc | Vital Signs Monitor | 73XT-B | 3.12% | $ 2,650.00 | [https://www.medstockusa.com/products/connex-bluetooth-spot-monitor-w-surebp-nibp-suretemp-plus-thermometer-73xt-b?variant=12402889654351&currency=USD](https://www.medstockusa.com/products/connex-bluetooth-spot-monitor-w-surebp-nibp-suretemp-plus-thermometer-73xt-b?variant=12402889654351&currency=USD&utm_medium=product_sync&utm_source=google&utm_content=sag_organic&utm_campaign=sag_organic&srsltid=ASuE1wQA3uzO3dQlAyUBwnpBwMYDpKP4pZRN3jM1DOuzp2VpVhFizTUWIHU) |  |  | $ 2,650.00 |
| Welch Allyn Inc | Vital Signs Monitor | Connex Spot | 2.91% | $ 2,604.00 | <https://medicalrite.com/products/connex_spot_monitor_6000_series_on_stand?variant=45612525814064> |  |  | $ 2,604.00 |
| Philips Healthcare | Multi-Parameter Module/Monitor | MX700 | 2.58% | $ 9,995.00 | <https://kenmedsurgical.com/products/philips-intellivue-mx700-icu-pacu-patient-monitoring-system-nibp-spo2-ecg-co2?currency=USD&variant=44412601041055> | $ 7,519.00 | <https://bimedis.com/philips-intellivue-mx-700-m261449> | $ 10,025.33 |
| Welch Allyn Inc | Vital Signs Monitor | Spot Vital Signs LXi | 2.25% |  |  | $ 1,417.00 | [https://mfimedical.com/products/welch-allyn-spot-lxi-vital-signs-monitor?variant=39595708678221&currency=USD](https://mfimedical.com/products/welch-allyn-spot-lxi-vital-signs-monitor?variant=39595708678221&currency=USD&utm_medium=product_sync&utm_source=google&utm_content=sag_organic&utm_campaign=sag_organic&gclid=CjwKCAjwkLCkBhA9EiwAka9QRi8hyTJH-ZZc9tEnV_1IbCrajenbLDnVaZp4J-XH-QDK6yjWReCa4xoCqk0QAvD_BwE) | $ 1,889.33 |
| GE Healthcare | Multi-Parameter Module/Monitor | PDM | 1.95% |  |  | $ 1,504.00 | <https://bimedis.com/ge-pdm-m477466> | $ 2,005.33 |
| Philips Healthcare | Multi-Parameter Module/Monitor | MX400 | 1.37% |  |  | $ 3,456.00 | <https://bimedis.com/philips-intellivue-mx400-m407885> | $ 4,608.00 |
| GE Healthcare | Vital Signs Monitor | V100 | 1.35% | $ 2,805.00 | [https://mfimedical.com/products/ge-carescape-v100-vital-signs-monitor?variant=31492852547661&currency=USD](https://mfimedical.com/products/ge-carescape-v100-vital-signs-monitor?variant=31492852547661&currency=USD&utm_medium=product_sync&utm_source=google&utm_content=sag_organic&utm_campaign=sag_organic&gclid=CjwKCAjw-7OlBhB8EiwAnoOEk-bzKSCV9SelxjxFxI2TfEz0nNa-43BvoXfQVVZOpIrm7wfr-0XZGhoCVWkQAvD_BwE) |  |  | $ 2,805.00 |
| Masimo Corp | Pulse Ox Module/Monitor | Rad-5 | 1.28% | $ 899.98 | <https://supplystorenow.com/products/masimo-rad-5-handheld-pulse-oximeter-9196> |  |  | $ 899.98 |
| Welch Allyn Inc | Vital Signs Monitor | Spot Vital Signs 420 | 1.17% |  |  | $ 1,417.00 | <https://mfimedical.com/products/welch-allyn-spot-lxi-vital-signs-monitor?variant=39595708612685&currency=USD> | $ 1,889.33 |
| GE Healthcare | Multi-Parameter Module/Monitor | Dash 3000 | 1.08% |  |  | $ 1,119.00 | <https://bimedis.com/ge-dash-3000-m4598> | $ 1,492.00 |
| Philips Healthcare | Pulse Ox Module/Monitor | Intellivue MMX | 1.05% |  |  |  |  | $ - |
