# Supplementary material for: The high price of equity in pulse oximetry: A cost evaluation and need for interim solutions
Source: PLOS Digit Health. 2024 Sep 30;3(9):e0000372. doi: 10.1371/journal.pdig.0000372 (PMC11441667; doi:10.1371/journal.pdig.0000372)
Supplement: S2 Table — (DOCX) [file pdig.0000372.s004.docx]

## S2 Table. Average replacement cost per bed

This table reflects the cost of replacing pulse oximetry equipment by bed size, along with a variation based on the price. This is based on the total cost of pulse oximetry replacement - for the 100% of the estimated price for the multiparameter models, 50% of single parameter models/modules, and 0% of vitals signs monitors. This estimate includes a mix of pulse ox monitors (which require replacing the entire monitor) and modules (which require replacing only the module). A 75% used price to new price ratio suggests that a hypothetical device that can be purchased used for $750 is estimated to have an MSRP of $1,000.

DUH: Duke University Hospital

DRaH: Duke Raleigh Hospital

DRH: Duke Regional Hospital

DUHS: Duke University Health System.

|  | **DUH** | **DRaH** | **DRH** | **DRaH + DRH** | **DUHS** |
| --- | --- | --- | --- | --- | --- |
|  | $ 6,811.12 | $ 12,772.34 | $ 3,975.10 | $ 6,902.27 | $ 6,834.61 |
| used percentage of new |  |  |  |  |  |
| 50% | $ 8,796.56 | $ 16,002.65 | $ 4,903.90 | $ 8,596.86 | $ 8,715.47 |
| 75% | $ 6,811.12 | $ 12,772.34 | $ 3,975.10 | $ 6,902.27 | $ 6,834.61 |
| 90% | $ 6,149.31 | $ 11,695.58 | $ 3,665.50 | $ 6,337.40 | $ 6,207.66 |
