# Supplementary material for: The high price of equity in pulse oximetry: A cost evaluation and need for interim solutions
Source: PLOS Digit Health. 2024 Sep 30;3(9):e0000372. doi: 10.1371/journal.pdig.0000372 (PMC11441667; doi:10.1371/journal.pdig.0000372)
Supplement: S3 Table — (DOCX) [file pdig.0000372.s005.docx]

## S3 Table. Device integration costs

Estimated costs for device integration at each hospital given the number of different devices. Costs in thousands.

|  | **Hospitals A, B, C** | | | **A** | | | **B** | | | **C** | | |
| --- | --- | --- | --- | --- | --- | --- | --- | --- | --- | --- | --- | --- |
|  | **n** | **250 hours** | **500 hours** | **n** | **250 hours** | **500 hours** | **n** | **250 hours** | **500 hours** | **n** | **250 hours** | **500 hours** |
| **Multi- Parameter Module /Monitor** | 48 | $ 2,400.00 | $ 4,800.00 | 39 | $ 1,950.00 | $ 3,900.00 | 24 | $ 1,200.00 | $ 2,400.00 | 13 | $ 650.00 | $ 1,300.00 |
| **Pulse Ox Module/ Monitor** | 34 | $ 850.00 | $ 1,700.00 | 28 | $ 700.00 | $ 1,400.00 | 9 | $ 225.00 | $ 450.00 | 5 | $ 125.00 | $ 250.00 |
| **Vital Signs Monitor** | 58 | $ - | $ - | 53 | $ - | $ - | 9 | $ - | $ - | 8 | $ - | $ - |
|  |  | $ 3,250.00 | $ 6,500.00 |  | $ 2,650.00 | $ 5,300.00 |  | $ 1,425.00 | $ 2,850.00 |  | $ 775.00 | $ 1,550.00 |
