# Supplementary material for: The high price of equity in pulse oximetry: A cost evaluation and need for interim solutions
Source: PLOS Digit Health. 2024 Sep 30;3(9):e0000372. doi: 10.1371/journal.pdig.0000372 (PMC11441667; doi:10.1371/journal.pdig.0000372)
Supplement: S4 Table — (DOCX) [file pdig.0000372.s006.docx]

## S4 Table. National estimates in fleet replacement cost, using different used/new price estimates, assuming average prices across health system

This table reflects the extrapolated cost of replacing pulse oximetry equipment for all 5,564 hospitals in the 2015 CDC hospital data chart. This model explores a range of costs for replacing pulse oximetry per bed from using the new price as 133% of the used price, and ranging from 111%-200% of the used price. A 75% used price to new price ratio suggests that a hypothetical device that can be purchased used for $750 is estimated to have an MSRP of $1,000. This does not include integration costs.

|  |  |  | **cost per hospital,**  **using 75%** | **extrapolated, 75%** | **cost per hospital, using 90%** | **extrapolated cost, 90%** | **cost per hospital,**  **using 50%** | **extrapolated cost, 50%** |
| --- | --- | --- | --- | --- | --- | --- | --- | --- |
| **Hospital type** | **n** | **beds per hospital** | **$ 6.83** |  | **$ 6.21** |  | **$ 8.72** |  |
| All hospitals | 5,564 | 161.39 | $ 1.10 | $ 6,137.21 | $ 1.00 | $ 5,574.23 | $ 1.41 | $ 7,826.15 |
| Federal | 212 | 183.32 | $ 1.25 | $ 265.61 | $ 1.14 | $ 241.25 | $ 1.60 | $ 338.71 |
| Nonfederal | 5,352 | 160.52 | $ 1.10 | $ 5,871.60 | $ 1.00 | $ 5,332.99 | $ 1.40 | $ 7,487.44 |
| Community | 4,862 | 160.88 | $ 1.10 | $ 5,345.95 | $ 1.00 | $ 4,855.56 | $ 1.40 | $ 6,817.14 |
| Nonprofit | 2,845 | 186.50 | $ 1.27 | $ 3,626.30 | $ 1.16 | $ 3,293.65 | $ 1.63 | $ 4,624.25 |
| For profit | 1,034 | 130.14 | $ 0.89 | $ 919.73 | $ 0.81 | $ 835.36 | $ 1.13 | $ 1,172.83 |
| State-local government | 983 | 119.06 | $ 0.81 | $ 799.92 | $ 0.74 | $ 726.54 | $ 1.04 | $ 1,020.06 |
| 6–24 beds | 499 | 16.51 | $ 0.11 | $ 56.30 | $ 0.10 | $ 51.13 | $ 0.14 | $ 71.79 |
| 25–49 beds | 1,146 | 32.30 | $ 0.22 | $ 253.02 | $ 0.20 | $ 229.81 | $ 0.28 | $ 322.65 |
| 50–99 beds | 916 | 71.19 | $ 0.49 | $ 445.67 | $ 0.44 | $ 404.79 | $ 0.62 | $ 568.32 |
| 100–199 beds | 983 | 144.93 | $ 0.99 | $ 973.73 | $ 0.90 | $ 884.41 | $ 1.26 | $ 1,241.70 |
| 200–299 beds | 535 | 245.40 | $ 1.68 | $ 897.30 | $ 1.52 | $ 814.98 | $ 2.14 | $ 1,144.23 |
| 300–399 beds | 322 | 345.15 | $ 2.36 | $ 759.59 | $ 2.14 | $ 689.91 | $ 3.01 | $ 968.63 |
| 400–499 beds | 177 | 442.24 | $ 3.02 | $ 534.99 | $ 2.75 | $ 485.91 | $ 3.85 | $ 682.21 |
| >= 500 beds | 284 | 734.33 | $ 5.02 | $ 1,425.36 | $ 4.56 | $ 1,294.61 | $ 6.40 | $ 1,817.61 |
