# Supplementary material for: The high price of equity in pulse oximetry: A cost evaluation and need for interim solutions
Source: PLOS Digit Health. 2024 Sep 30;3(9):e0000372. doi: 10.1371/journal.pdig.0000372 (PMC11441667; doi:10.1371/journal.pdig.0000372)
Supplement: Attachment — Submitted filename: PDIG-D-23-00357 - response to editorial review.docx [file pdig.0000372.s007.docx]

*PDIG-D-23-00357*

*The High Price of Equity in Pulse Oximetry: A cost evaluation and need for interim solutions*

*PLOS Digital Health*

*Dear Dr. Wong,*

*Thank you for submitting your manuscript to PLOS Digital Health. After careful consideration, we feel that it has merit but does not fully meet PLOS Digital Health's publication criteria as it currently stands. Therefore, we invite you to submit a revised version of the manuscript that addresses the points raised during the review process.*

*Please submit your revised manuscript within 60 days May 05 2024 11:59PM. If you will need more time than this to complete your revisions, please reply to this message or contact the journal office at digitalhealth@plos.org. When you're ready to submit your revision, log on to https://www.editorialmanager.com/pdig/ and select the 'Submissions Needing Revision' folder to locate your manuscript file.*

*Please include the following items when submitting your revised manuscript:*

** A rebuttal letter that responds to each point raised by the editor and reviewer(s). You should upload this letter as a separate file labeled 'Response to Reviewers'.*

** A marked-up copy of your manuscript that highlights changes made to the original version. You should upload this as a separate file labeled 'Revised Manuscript with Track Changes'.*

** An unmarked version of your revised paper without tracked changes. You should upload this as a separate file labeled 'Manuscript'.*

*If you would like to make changes to your financial disclosure, please include your updated statement in your cover letter. Guidelines for resubmitting your figure files are available below the reviewer comments at the end of this letter.*

*We look forward to receiving your revised manuscript.*

*Kind regards,*

*Nan Liu*

*Academic Editor*

*PLOS Digital Health*

*Journal Requirements:*

*1. Please send a completed 'Competing Interests' statement, including any COIs declared by your co-authors. If you have no competing interests to declare, please state "The authors have declared that no competing interests exist". Otherwise please declare all competing interests beginning with the statement "I have read the journal's policy and the authors of this manuscript have the following competing interests:"*

Thank you for your comment. The manuscript contains the following statement to declare no competing interests exist; “The other authors have no conflicts of interest to disclose.”

**Funding**

AIW is supported by the Duke CTSI by the National Center for Advancing Translational Sciences of the NIH under UL1TR002553 and the National Institute on Minority Health and Health Disparities REACH Equity Award under 5U54MD012530. All others state no conflict of interest.

The ~~other authors have no conflicts of interest to disclose~~funders had no role in study design, data collection and analysis, decision to publish, or preparation of the manuscript.

*2. Please amend your detailed Financial Disclosure statement. This is published with the article. It must therefore be completed in full sentences and contain the exact wording you wish to be published.*

*a. State the initials, alongside each funding source, of each author to receive each grant.*

*b. State what role the funders took in the study. If the funders had no role in your study, please state: “The funders had no role in study design, data collection and analysis, decision to publish, or preparation of the manuscript.”*

*c. If any authors received a salary from any of your funders, please state which authors and which funders.*

*If you did not receive any funding for this study, please simply state: “The authors received no specific funding for this work.*

Thank you for your comment. The initials of the author who received the funding is provided. Additionally, we disclosed that the funders did not have a role in the design, data collection and analysis, decision to publish, or preparation of the manuscript and did not receive a salary.

**Funding**

AIW is supported by the Duke CTSI by the National Center for Advancing Translational Sciences of the NIH under UL1TR002553 and the National Institute on Minority Health and Health Disparities REACH Equity Award under 5U54MD012530. All others state no conflict of interest.

The ~~other authors have no conflicts of interest to disclose~~funders had no role in study design, data collection and analysis, decision to publish, or preparation of the manuscript.

*3. Please provide separate figure files in .tif or .eps format only and remove any figures embedded in your manuscript file. Please also ensure that all files are under our size limit of 10MB.*

*For more information about figure files please see our guidelines:*

[*https://journals.plos.org/digitalhealth/s/figures*](https://journals.plos.org/digitalhealth/s/figures)

[*https://journals.plos.org/digitalhealth/s/figures#loc-file-requirements*](https://journals.plos.org/digitalhealth/s/figures#loc-file-requirements)

Thank you for your comment. We ensured that the figure files meet the journal requirements. Note that we have revised Table 1 to include images of devices for clarity.

*4. We have noticed that you have uploaded Supporting Information files, but you have not included a list of legends. Please add a full list of legends for your Supporting Information files after the references list.*

Thank you for your comment. We have added a “Supporting Information Files” section at the end of the document, listing the supplement tables provided.

## *Supporting Information Files*

*Supplemental Table 1. List of price estimates for devices*

*Supplemental Table 2. Average replacement cost per bed.*

*Supplemental Table 3. Device integration costs*

*Supplemental Table 4. National estimates in fleet replacement cost, using different used/new price estimates, assuming average prices across health system*

*Additional Editor Comments (if provided):*

*We would like to thank the authors for bringing out an important issue for discussions. While the topic of the study is interesting, there lacks sufficient details and background information to justify its scientific rigor. The authors are suggested to elaborate on various sections of the manuscript to ensure solid methods and convincing results.*

Thank you for your comment. We have added significantly more detail to the background, results, and discussion sections to further signify scientific rigor to help ensure understanding and reproducibility of the methods and results. We have also added a table to the background (Table 1) for transparency of devices investigated and modified Table 2 to disclose the hospital system analyzed as well as provide insight of the 3 individual hospitals of focus (i.e. academic centers versus community hospitals).

*[Note: HTML markup is below. Please do not edit.]*

*Reviewers' comments:*

*Reviewer's Responses to Questions*

***Comments to the Author***

*1. Does this manuscript meet PLOS Digital Health’s* [*publication criteria*](https://journals.plos.org/digitalhealth/s/journal-information#loc-criteria-for-publication)*? Is the manuscript technically sound, and do the data support the conclusions? The manuscript must describe methodologically and ethically rigorous research with conclusions that are appropriately drawn based on the data presented.*

*Reviewer #1: Yes*

*Reviewer #2: Yes*

*Reviewer #3: No*

*--------------------*

*2. Has the statistical analysis been performed appropriately and rigorously?*

*Reviewer #1: Yes*

*Reviewer #2: Yes*

*Reviewer #3: No*

*--------------------*

*3. Have the authors made all data underlying the findings in their manuscript fully available (please refer to the Data Availability Statement at the start of the manuscript PDF file)?*

*The* [*PLOS Data policy*](https://journals.plos.org/digitalhealth/s/data-availability) *requires authors to make all data underlying the findings described in their manuscript fully available without restriction, with rare exception. The data should be provided as part of the manuscript or its supporting information, or deposited to a public repository. For example, in addition to summary statistics, the data points behind means, medians and variance measures should be available. If there are restrictions on publicly sharing data—e.g. participant privacy or use of data from a third party—those must be specified.*

*Reviewer #1: Yes*

*Reviewer #2: Yes*

*Reviewer #3: No*

*--------------------*

*4. Is the manuscript presented in an intelligible fashion and written in standard English?*

*PLOS Digital Health does not copyedit accepted manuscripts, so the language in submitted articles must be clear, correct, and unambiguous. Any typographical or grammatical errors should be corrected at revision, so please note any specific errors here.*

*Reviewer #1: Yes*

*Reviewer #2: Yes*

*Reviewer #3: Yes*

*--------------------*

## Reviewer 1

*5. Review Comments to the Author*

*Please use the space provided to explain your answers to the questions above. You may also include additional comments for the author, including concerns about dual publication, research ethics, or publication ethics. (Please upload your review as an attachment if it exceeds 20,000 characters)*

*Reviewer #1: This is a well formulated paper. The findings that “Simply replacing” pulse oximetry hardware to address accuracy disparities may be neither simple, cheap, or timely, and that solutions for addressing pulse oximetry accuracy disparities leveraging current technology may be necessary, are of value to informing current and future service provision.*

Thank you for your comment.

## Reviewer 2

*Reviewer #2: This is an interesting article that extrapolates the expense of replacing pulse oximetry hardware and software across 1 hospital system and the nation itself highlighting the depth of the issue and difficulty in rectifying.*

*I have a few comments and consideration for revision for the authors:*

*Intro*

*-The introduction may benefit from a little more description/explanation of the differences between the different models and what the differences are in terms of considerations when replacing. For example, why are pulse ox modules easier to replace than vital signs monitors*

Thank you for your comment. To provide more description/explanation of the differences between the different models and considerations (characterized as ease of replacement), we have added a table, Table 1. Additionally, we have further addressed why pulse ox modules are easier to replace compared to vital sign monitors and we have found that it is due to the fact that standalone devices require manual date entry.

*Pulse oximetry ~~devices fall~~equipment falls into three categories:*

*~~●~~*	***~~Multi-Parameter Models~~*** *multi-parameter models (e.g~~.~~, Philips MX800, GE Dash 4000~~): Complex~~, Philips X3) are complex units that display multiple vital signs, predominantly used in ICUs and operating rooms~~.~~*

*~~●~~*	***~~Pulse Ox~~****; Pulse Oximetry Modules/Monitors (e.g~~.~~, Masimo Rad-7)~~: Specialized~~ are specialized for pulse oximetry~~, these~~ and can be integrated into existing systems without replacing whole units~~.~~*

*~~●~~	; and Vital Signs Monitors (e.g~~.~~, Philips SureSigns VS4)~~: Primarily~~ are primarily used in outpatient and general inpatient settings, ~~these collect~~collecting various vital signs~~.~~*

*Methods*

*-it is unclear to me from the methods, exactly what technology would be considered to replace the current pulse ox monitors. My (limited) understanding was* ***that we did not have pulse ox monitors that would overcome the skin color difficulties.*** *But I may be mistaken. When discussing the potential of which equipment was used to calculate costs, it is a little unclear to me what actual equipment/modules/monitors are being considered. If no specific module or monitor is being considered, then I would be interested if the paper more explicitly stated that the authors extrapolated cost of replacement using existing monitor/module equipment models and prices*

Thank you for your comment. At this time, there is evidence showing how current pulse oximeters are limited to skin tone variations and this was further described in the background section by discussing concerns of the FDA and the role of stakeholders. Additionally, specific examples of equipment/modules/monitors considered are provided in a table in the background section (Table 1).

### Device integration estimates

The EHR integration process involves multiple ~~variables~~steps, including HL7 interface, licenses, server specifications, firewalls, and software revisions. ~~The time required was estimated at~~Estimates on integration time required by Duke Performance Services leadership, which oversees device integration, recommended 250-500 person-hours per device model, at a rate of $200/hour. Costs were fully applied to multi-parameter monitors, 50% to pulse ~~ox~~oximetry modules/monitors, and not applied to vital signs monitors.

*-It may help the less technologically aware reader, what exactly is meant by the device "integration process." Is this integration with the EMR, computer system, telemetry monitoring/alarm system. All of the above? none of the above?*

Thank you for your comment. We have further explained the device integration process by clarifying that this is EMR integration only, hence why no integration costs were for vital signs monitors (e.g., Dinamap). We do note in Table 1 that some newer vital signs monitors (e.g., Phillips EarlyVue) can be integrated in the EHR, but then this will add integration costs.

### Device integration estimates

The EHR integration process involves multiple ~~variables~~steps, including HL7 interface, licenses, server specifications, firewalls, and software revisions. ~~The time required was estimated at~~Estimates on integration time required by Duke Performance Services leadership, which oversees device integration, recommended 250-500 person-hours per device model, at a rate of $200/hour. Costs were fully applied to multi-parameter monitors, 50% to pulse ~~ox~~oximetry modules/monitors, and not applied to vital signs monitors.

*-I am not sure I understand what is meant by "fleet" is fleet the collection of pulse ox monitors/modules across a hospital system?*

Thank you for your comment. To limit the use of technical language, the term “fleet” has been modified to “total”.

*-Overall I think the methods was written for someone with technological understanding of what replacement of any hospital system includes and may benefit from revision based on above thoughts*

Thank you for your comment. We have further explained the integration process regarding replacement of current pulse oximeter devices.

### Device integration estimates

The EHR integration process involves multiple ~~variables~~steps, including HL7 interface, licenses, server specifications, firewalls, and software revisions. ~~The time required was estimated at~~Estimates on integration time required by Duke Performance Services leadership, which oversees device integration, recommended 250-500 person-hours per device model, at a rate of $200/hour. Costs were fully applied to multi-parameter monitors, 50% to pulse ~~ox~~oximetry modules/monitors, and not applied to vital signs monitors.

*Results:*

*-Under cost estimates for a single health system, would the authors be able to explain what "specialized care" means - what are the 332 specialized care beds? are they ICU beds?*

Thank you for your comment. We utilized CMS to provide further clarification and to define specialized care.

### Cost estimates for a single academic health system

~~Hospital System X comprises three main facilities. Hospital A is a large center with 1,048 beds, including 332 designated for specialized care. Hospital B~~Duke University Health System (DUHS) is comprised of three main facilities. Duke University Hospital (DUH) is a large, quaternary, academic center with 1,048 beds, including 332 designated for specialized care (i.e., defined as a hospital bed in which special medical/surgical services, beyond general medical/surgical care and including intensive care or coronary care, are provided. [[18]](https://paperpile.com/c/ot0igV/bte7)). Duke Raleigh Hospital (DRaH) is a medium-sized facility with 186 beds, of which 28 are specialized. ~~Hospital C~~Duke Regional Hospital (DRH) has 373 beds, with 22 allocated for specialized care.

*-The results describe that there are 140 types of pulse ox equipment - in the intro the authors describe 3 types. what is meant by 140 distinct types? distinct companies? Seems surprising to me that one hospital system would have so many types of pulse ox*

Thank you for your comment. The 140 distinct models of pulse oximeter equipment are based on multi-parameter module/monitor, pulse ox module/monitor, and vital signs monitors across all three hospitals in Duke’s Health System (Duke Raleigh, Duke Regional, and Duke University Hospital). The distinct types are described in Supplemental Table 1 for all devices over 1% incidence

*-missing in results is a discussion of the time it would take for replacement. Understanding that is not the aim of the study, the discussion talks about the time to replacement adn would be curious if the authors had any information on estimating time to replacement*

Thank you for your comment. This is a complicated point, noting that there is procurement time (unmeasured), integration time (estimated at 250-500 hours by Duke Performance Services, who integrates the equipment). However, some hospitals may decide to initiate these processes only after equipment has reached end of life. Some devices (e.g., Dinamap) have been in use since 2008 and continue to be in use.

*Table 2:*

*This is a minor point and may not apply to many, but I was a little confused trying to interpret what $20,000 (in millions) refers to. Is there a better way to present the cost in this table?*

Thank you for your comment. Tables have now been aligned to be all thousands or millions.

## Reviewer 3

*Reviewer #3: This is an observational cross-evaluation of the cost of a pulse oximetry device and its replacement costs. As a report, I see the value in this type of information. However, it is questionable what this adds to the value of new information. The paper does not reveal the health system the analysis was carried out, and I am unsure if this is questionable as results cannot be replicated or checked elsewhere.*

*The background provides scant details of why this study is needed. It briefly touches on the racial disparities of pulse oximetry but does not elaborate on the justification of how the results could be used in a policy setting to reduce such issues.*

*I believe this provides some information as a report to the government or policymakers, but I cannot see the rigor or justification to be presented as a scientific paper.*

Thank you for your comment. After amendment with the IRB, the health system has been revealed; Duke University Health System, spanning Duke University Hospital, Duke Raleigh Hospital, and Duke Regional Hospital.

### Cost estimates for a single academic health system

~~Hospital System X comprises three main facilities. Hospital A is a large center with 1,048 beds, including 332 designated for specialized care. Hospital B~~Duke University Health System (DUHS) is comprised of three main facilities. Duke University Hospital (DUH) is a large, quaternary, academic center with 1,048 beds, including 332 designated for specialized care (i.e., defined as a hospital bed in which special medical/surgical services, beyond general medical/surgical care and including intensive care or coronary care, are provided. [[18]](https://paperpile.com/c/ot0igV/bte7)). Duke Raleigh Hospital (DRaH) is a medium-sized facility with 186 beds, of which 28 are specialized. ~~Hospital C~~Duke Regional Hospital (DRH) has 373 beds, with 22 allocated for specialized care. Across these hospitals, 140 distinct ~~types~~models of pulse oximetry equipment are in use~~. Available pricing data cover 85.9% of all 8,460 devices in the system, and 67.1% of these are actively in use within the hospitals~~ (as seen in Table 2). Of all 8,460 devices at Duke University Health System (DUHS), 5,678 (67.1%) devices are used in hospitals. 1,542 of these devices are multiparameter monitors that have their pulse oximetry hardware in external monitors or modules (e.g., MX450, MX800 as the multiparameter monitor, relying on the Phillips X2 or X3 multiparameter monitor/module for pulse oximetry hardware. See Table 1 for examples).

Furthermore, the background has been revised and provides further detail of racial disparities of pulse oximetry and impact on policy.

## *Background*

*Pulse oximetry is a critical tool in modern medicine~~.~~*[*^1^*](https://paperpile.com/c/BvsZYL/SnaZ) *~~Patients of color - especially Black patients - have been associated with increased~~ that provides a continuous and real-time measurement of arterial oxygenation, a critical marker of respiratory function.*[*[1]*](https://paperpile.com/c/ot0igV/XeKEO) *However, in patients with dark skin pigmentation – especially Black patients – pulse oximetry accuracy disparities have been demonstrated, as evidenced by increased hidden or occult hypoxemia~~.~~*[*^2,3^*](https://paperpile.com/c/BvsZYL/ifkG+VorQ)*, which was first identified in December 2020.*[*[2,3]*](https://paperpile.com/c/ot0igV/yY4zJ+3iIaf) *These inaccuracies are associated with increased mortality, organ dysfunction, decreased oxygen therapy, and delayed COVID recognition.*[*[*](https://paperpile.com/c/ot0igV/TCfVL+3iIaf+O0dEk)[*3–5*](https://paperpile.com/c/BvsZYL/F1jP+VorQ+nCls)[*3–5*](https://paperpile.com/c/ot0igV/TCfVL+3iIaf+O0dEk) *~~Many have called for simply replacing all pulse oximetry devices with new technology. There are early possibilities, such as green light oximetry, cameras, and improved algorithms.~~*[*^6–8^*](https://paperpile.com/c/BvsZYL/boGx+MrQT+0wlP)[*]*](https://paperpile.com/c/ot0igV/TCfVL+3iIaf+O0dEk)

*In February 2021, the Food and Drug Administration (FDA) issued a safety communication calling on patients and health care providers to be aware that pulse oximeters have critical limitations.*[*[6]*](https://paperpile.com/c/ot0igV/cKa1) *Many have called for simply replacing all pulse oximetry devices with new technology; as a consequence, the FDA is engaging with multiple stakeholders to reassess existing pulse oximetry clearance guidelines.* [*[7,8]*](https://paperpile.com/c/ot0igV/mcbz+Sfqn) *There are early possibilities, such as green light oximetry, cameras, and improved algorithms,*[*[9–11]*](https://paperpile.com/c/ot0igV/OzZcb+7MFg3+qWXkW) *that are supported by alternative physical principles to estimate arterial oxygen saturation in a way that is not sensitive to skin pigmentation. These novel devices will likely require new equipment for both pulse oximetry probes and monitors. However, “simple replacement” of existing devices may ~~neither~~not be simple, cheap, nor timely. ~~The objective of this manuscript is to describe the financial and logistical burden in replacing pulse oximeters at one health system and across the United States.~~* [*^2,6^*](https://paperpile.com/c/BvsZYL/ifkG+boGx)

*~~Types of pulse oximetry equipment~~* [*[12]*](https://paperpile.com/c/ot0igV/8qr2) *Flawed pulse oximeters are still ubiquitously used at present, including home, ambulatory, and hospital settings.*[*[13]*](https://paperpile.com/c/ot0igV/F3yf) *Moreover, pulse oximeters are often integrated into hospital-wide complex electronic health records (EHR) systems; current medical device ecosystems often require vendor-specific interfaces that hamper interoperability and integration of new devices.* [*[14]*](https://paperpile.com/c/ot0igV/iC23)

*--------------------*

*6. PLOS authors have the option to publish the peer review history of their article (*[*what does this mean?*](https://journals.plos.org/digitalhealth/s/editorial-and-peer-review-process#loc-peer-review-history)*). If published, this will include your full peer review and any attached files.*

***Do you want your identity to be public for this peer review?*** *If you choose “no”, your identity will remain anonymous but your review may still be made public.*

*For information about this choice, including consent withdrawal, please see our* [*Privacy Policy*](https://www.plos.org/privacy-policy)*.*

*Reviewer #1: Yes: Dr Sarah Markham*

*Reviewer #2: No*

*Reviewer #3: No*

*--------------------*
